# How language affects children's use of derivational morphology in visual word and pseudoword processing: evidence from a cross-language study

**DOI:** 10.3389/fpsyg.2015.00452

**Published:** 2015-04-16

**Authors:** Séverine Casalis, Pauline Quémart, Lynne G. Duncan

**Affiliations:** ^1^SCALab, Université de Lille and Centre National de la Recherche ScientifiqueVilleneuve d'Ascq, France; ^2^University of Poitiers and Centre National de la Recherche ScientifiquePoitiers, France; ^3^School of Psychology, University of DundeeDundee, UK

**Keywords:** morphology, reading acquisition, cross language comparison, visual word recognition, lexical decision task

## Abstract

Developing readers have been shown to rely on morphemes in visual word recognition across several naming, lexical decision and priming experiments. However, the impact of morphology in reading is not consistent across studies with differing results emerging not only between but also within writing systems. Here, we report a cross-language experiment involving the English and French languages, which aims to compare directly the impact of morphology in word recognition in the two languages. Monolingual French-speaking and English-speaking children matched for grade level (Part 1) and for age (Part 2) participated in the study. Two lexical decision tasks (one in French, one in English) featured words and pseudowords with exactly the same structure in each language. The presence of a root (R+) and a suffix ending (S+) was manipulated orthogonally, leading to four possible combinations in words (R+S+: e.g., postal; R+S−: e.g., turnip; R−S+: e.g., rascal; and R-S-: e.g., bishop) and in pseudowords (R+S+: e.g., pondal; R+S−: e.g., curlip; R−S+: e.g., vosnal; and R−S−: e.g., hethop). Results indicate that the presence of morphemes facilitates children's recognition of words and impedes their ability to reject pseudowords in both languages. Nevertheless, effects extend across accuracy and latencies in French but are restricted to accuracy in English, suggesting a higher degree of morphological processing efficiency in French. We argue that the inconsistencies found between languages emphasize the need for developmental models of word recognition to integrate a morpheme level whose elaboration is tuned by the productivity and transparency of the derivational system.

## Introduction

In a recent paper, Frost (2012) has put forward a case for a universal model of reading. As it is not certain that, as a cultural product, written language should be subject to a universal form of processing (Coltheart and Crain, 2012), it seems important to consider whether variations in language properties constrain the use of particular language units. Deacon (2012) rightly pointed out the relevance of the developmental approach to deal with this issue since a key aspect of developmental studies is that they tell us which skills drive reading acquisition and which are the product of reading. In other words, developmental cross language studies should help to disentangle which aspects of reading acquisition are universal and which depend on language properties. Therefore, the aim of the present paper is to compare how English-speaking and French-speaking developing readers make use of one of the fundamental units of reading development, namely, morphemes.

Research conducted over three decades has documented the importance of phonological coding in the earliest phases of learning to read an alphabetic script (Goswami and Bryant, 1990; Muter et al., 2004; Melby-Lervåg et al., 2012). The key unit relevant for phonological coding in alphabetic scripts is the grapheme and its oral counterpart, the phoneme. However, spelling-to-sound consistency varies across orthographies (Frost et al., 1987) and alphabetic orthographies are distinguished along a continuum from transparent to opaque. In some orthographies, grapheme-phoneme correspondences (hereafter, GPC) are transparent, with individual graphemes always pronounced in the same way (e.g., Finnish, Italian). In other orthographies, GPC are highly opaque, meaning that the same grapheme can be pronounced in different ways (e.g., English). The French orthography is opaque in terms of spelling, indeed similar to English in this respect, but more transparent when it comes to reading.

Orthographic transparency has an impact on the ease with which children learn to read across countries, and reading achievement at the end of the first year clearly depends on the consistency of the GPC (Seymour et al., 2003; Duncan et al., 2013). Learning to read is particularly difficult for English-speaking children, who perform at a much lower level than children learning to read in other languages. This delay is observed when reading both familiar words and pseudowords in the initial phases of reading acquisition (Seymour et al., 2003). The Psycholinguistic Grain-Size theory (PGST, Ziegler and Goswami, 2005) has been proposed to account for the effects of such cross-language differences in orthographic depth on reading acquisition (Ziegler et al., 2001; Ziegler and Goswami, 2006). This model suggests that reading development across alphabetic scripts may display some variation in the grain size that children utilize as a function of the availability of units in oral language and the consistency of the links between these units of speech and written orthographic symbols. Even though the PGST focuses on reading aloud, some features may generalize to other aspects of reading such as silent visual word recognition. Equally, other written units such as morphemes, which are not included in this model may come to play a role during literacy acquisition, particularly if these units are available in language and resolve irregularity within the orthography (Ziegler et al., 1997).

The role of morphology in learning to read alphabetic scripts has received increased attention over the past two decades due to a number of factors: (1) most alphabetic writing systems are morphophonemic, in that they represent both phonemic and morphemic units; (2) the majority of new words that children encounter in print are morphologically complex (Nagy and Anderson, 1984), which means that decomposing complex words into smaller constituents during visual word recognition should be particularly relevant when learning to read these words; and (3) developing readers have acquired morphological awareness of spoken language and represent morphological information within their lexicon (Duncan et al., 2009), so given the “intimate relationship between spoken and written language skills” (Hulme and Snowling, 2013, p. 1), word reading is likely to draw upon this ability, particularly in the case of morphologically complex words.

The role of morphology in children's visual word processing has been examined across several languages. In English, children name derived words (e.g., *dancer*) more accurately than pseudoderived words (e.g., *dinner*) as early as Grade 2 (Laxon et al., 1992; Carlisle and Stone, 2005). This effect depends on family size, i.e. the number of derived forms (Carlisle and Katz, 2006), and on base word frequency for reading accuracy (Mann and Singson, 2003; Carlisle and Stone, 2005) and reading speed (Deacon et al., 2011). In Italian, third and fifth graders read pseudowords made up of morphemes (e.g., *donnista*) faster than control pseudowords (e.g., *donnosto*) (Burani et al., 2002, 2008). In relation to words, Italian children read derived words faster than non-derived words but this effect is limited to low frequency words (Marcolini et al., 2011). Finally, the presence of a base and/or a suffix facilitates visual word recognition in the French language (Quémart et al., 2012). When combined, such units also slow down lexical decisions, give rise to a high false alarm rate (Quémart et al., 2012) and enhance speed and accuracy of pseudoword naming (Colé et al., 2012).

Together, these results strongly support the importance of morphemes for developing readers when reading and/or accessing the lexicon but fail to provide a unified picture of the conditions under which this facilitation occurs, since the effects of morphological structure were not consistent. First, morphological structure significantly influenced both accuracy and latencies in French but was significant for accuracy only in English. Second, morphemes affected reading and lexical access when embedded in words and pseudowords in French, whereas such effects were observed only when morphemes were located within words in English and within pseudowords in Italian, except when words were low in frequency. Third, grade level or age of the participants was not constant across studies and there is reason to believe that the contribution of morphology to word processing is not the same during the first steps of reading acquisition as it is later when decoding mechanisms are well developed and more automatic. Finally, at least two different tasks have been used in previous studies, naming and lexical decision, complicating comparisons. Thus, to shed light on how language affects the use of morphology, cross-language studies using equivalent stimuli, a similar procedure and children at comparable grade or age levels are necessary.

To achieve this goal, the present study compares sensitivity to morphemes during visual word recognition among children speaking French vs. English. The French language is acknowledged as a morphologically rich language, with approximately 75% of French words being morphologically complex (Rey-Debove, 1984), while in English, morphologically complex (derived) forms account for 55% of the lemmas in the CELEX English database. Compounding is more prevalent in English than French, and is not especially productive in French especially in colloquial speech, thus word formation relies far more on derivation than compounding (Clark, 1998; Bauer, 2003). Indeed, French children perform higher in derived form production than English children (Duncan et al., 2009). In the present study, therefore, two effects may act in opposing directions on the outcome: first, the higher prevalence of affixes in French may make French readers more sensitive to this unit; and second, the depth of the English orthography, which makes GPC less reliable, may in turn favor the use of morphemes to increase the efficiency of English reading.

A key aspect of our study was to provide direct comparisons of how language and orthography impose variations in the use of morphemes in word recognition. This would contribute information about linguistic variation that would be useful in extending reading acquisition models to the morphological level. Our participants are typical readers in Grades 3 and 4, in other words, children who have already established early decoding in learning to read and who are expected to show morphemic effects on the basis of previous literature. However, we expect a degree of disparity between the groups due to cross-linguistic differences in relevant factors. The nature of these differences should help in understanding the impact of linguistic variation. We expect that orthographic depth and morphological productivity/transparency will both be influential. More use of morphemes would therefore be expected among the French group on the basis of morphological prevalence/ transparency but the question of whether the utility of morphemes in resolving the greater inconsistency in English will increase morphemic sensitivity in the English group beyond the level expected by the influence of morphological productivity/transparency has still to be resolved. In sum, if the presence of morphemes facilitates children's word recognition and if cognitive processing adapts to properties of environment stimuli, our first hypothesis is that children will rely on morphemic units when they process words and pseudowords, and our second hypothesis is that such morphological effects will be greater in the French language.

## Method

### Participants

Participants were 40 fourth graders from Scotland in the UK and 32 fourth graders from France. Both groups came from a similar middle income socioeconomic intake. The schools that we chose had middle-class catchment areas, according to national statistics in each country. Informed consent was obtained for each child. Mean age in the UK group was 8.41 years and mean age in the French group was 9.83 years. The difference was significant in terms of age (see Table 1) and not in terms of schooling because UK children start primary school 1 year before French children. A group of 32 French third graders matched for chronological age with the UK children was also recruited (see Table 1).

**Table 1:** **Characteristics of the participants: mean chronological and reading age in years(range in brackets)**.

	**English 4th gr**	**French 4th gr**	**French 3rd gr**	**En 4th gr-Fr 4th gr Student t**	***p*-value**	**En 4th gr-Fr 3th gr Student t**	***p*-value**
N	40	32	32				
Chronological age	8.41 (7.58–9.25)	9.83 (9.33–10.58)	8.67 (7.58–9.25)	15.19	<0.001	0.95	0.21
Reading level	9.58 (6.5–14)	9.83 (8.5–11.83)	9.16 (7.67–10.91)	0.82	0.42	1.07	0.029

*En, English; Fr, French; Gr, grade*.

### Background measures

To ensure that the two groups of fourth graders were comparable in terms of language abilities, we assessed receptive vocabulary in each group using the *British Picture Vocabulary Scale* in the United Kingdom (Dunn et al., 1997) and the *Echelle de Vocabulaire en Images Peabody* in France (Dunn et al., 1993). All children performed within the normal range (percentiles 25–90). Reading skills were assessed using the *British Ability Scales Word Reading* subtest (Elliott et al., 1983) in the United Kingdom and the *Alouette Test* (Lefavrais, 1967) in France. All children performed within the normal range (percentiles 25–90). The UK group displayed a reading age greater than their chronological age (see Table 1).

### Stimuli

A lexical decision task (LDT) was constructed following the same principles in both languages with close matching of stimuli for frequency, length and suffixes. We used the French Manulex database (Lété et al., 2004) and the English Children's Printed Word Database (CPWD, Masterson et al., 2003). There were four categories of words resulting from the presence or absence of a root and a suffix: (i) R+S+ [root and suffix, e.g., farmer (English), fermier [farmer] (French)]; (ii) R+S− [root but no suffix, e.g., window (English), boutique [shop] (French)]; (iii) R−S+ [no root but an (orthographic) suffix, e.g., murder (English), ménage [household] (French)]; and (iv) R−S− [no root and no suffix, e.g., narrow (English), pédale [pedal] (French)]. The items in condition (i) were the only real derivations. Pseudowords were formed from a similar principle resulting in four matched categories: (i) R+S+ [e.g., gifter (English), rosage (French)]; (ii) R+S− [e.g., puffow (English), lionque (French)]; (iii) R−S+ [e.g., gopter (English), mivage (French)]; and (iv) R−S− [e.g., ferbow (English), beadle (French)].

There were 29 items per condition in each language (see Appendix in Supplementary Material). Stimuli characteristics are presented in Table 2. There were 232 items in total. No fillers were added due to the length of the list. While this could potentially lead to an overestimation of the presence of embedded morphemes, our own assessment of the written language encountered by French children (via the Manulex database) indicates a high proportion of morpheme-like units. Due to differences in language characteristics, the roots in English derived words are nearly always complete, while roots are often truncated in French derived words. For example, the English word *farmer* contains the whole word root *farm*, while in the French word *fermier* [farmer], the final *e* of the root *ferme* has been removed. In our stimulus list, the whole lexical form of the root is truncated in 17 English words (10 in the R+S+ condition, 7 in the R+S− condition) and in 33 French words (22 in the R+S+ condition, 11 in the R+S- condition). In addition, in English, the base word is sometimes modified in the derived form by doubling the consonant. This is a peculiarity of English that complicates the orthographic definitions. However, given that this feature is quite common, it was also included as it was considered important to choose examples that were representative of the two languages in order to avoid concerns that our list of stimuli might be artificial in composition.

**Table 2:** **Stimuli characteristics in English and French languages**.

**WORDS**	**English**	**French**	**Difference student t**	***P*-value**
**R+S+**				
Frequency	46.83	41.46	0.27	ns
Length	6.38	7.10	2.99	0.01
R+: word frequency	106.14	156	1.16	ns
R+: length	4.21	5	4.3	0.01
**R+S−**				
Frequency	57.24	46.50	0.43	ns
Length	5.83	6.64	3.34	0.01
R+: word frequency	151.69	73.73	1.82	0.07
R+: length	3.72	3.89	0.75	ns
**R−S+**				
Frequency	59.97	45.49	0.81	ns
Length	6.241	6.59	1.33	ns
**R−S−**				
Frequency	44.55	44.38	0.04	Ns
Length	5.97	6.41	2.12	0.04
**PSEUDOWORDS**				
R+S+ length	6.35	7.04	2.55	0.01
R+S− length	5.83	6.79	4.41	0.00
R−S+ length	6.24	6.31	0.30	ns
R−S− length	5.97	6.10	0.61	ns

### Procedure

The lexical decision task was administered using Cognitive Workshop software (Seymour, 1994–1999) in the UK and E-prime Software, Version 1.0 (Schneider et al., 2002). Items were presented centrally in lower case Courier New font, size 25. The participants were required to press the “YES” key (using their dominant hand) if the string was a real word, and a “NO” key (using the non-dominant hand) if the string was not a real word. A trial consisted of a fixation cross during 1500 ms and the target remained on the screen until the participant responded or for a maximum of 5000 ms. Reaction times were recorded via the keyboard. There were two counterbalanced sessions with 6 practice items. Items were presented in a randomized order for each participant. All items categories were mixed within one list. A short pause was introduced after every 20 items.

## Results part I: grade-level matched comparison

### Data analysis

Due to differences in the age of schooling, UK children in Grade 4 were a year younger than their French counterparts. Therefore, we decided to conduct the analyses in two parts. We first compared the performances of the UK group to those of the French children matched for grade, and then we compared the performances of the UK group to those of the French group matched for age.

Analyses of variance were performed on percentages of correct responses (accuracy) and reaction times to correct responses, with root (R+, R−) and suffix (S+, S−) as within-subjects factors and language group (UK, French) as between−subjects factors. Only responses longer than 400 ms and shorter than 5000 ms were considered in the analysis (0.2% of the data were discarded from the analysis). We conducted analyses by participants (*F*_1_) and by items (*F*_2_) and for the sake of clarity, only significant (or marginally non-significant) effects—at least on *F*_1_ analyses—are reported.

### Word condition

#### Accuracy

**Figure 1:**
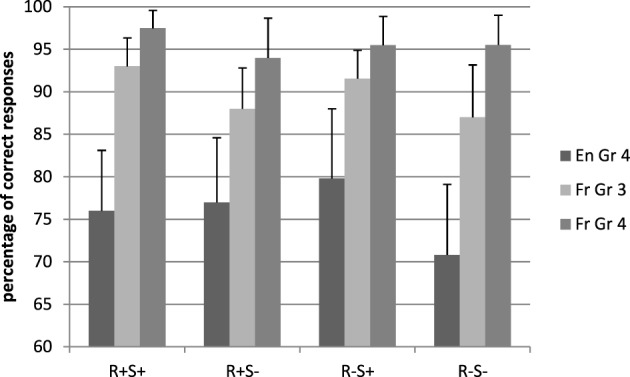
**Mean percentage of correct responses in the lexical decision task, real word conditions**. En, English; Fr, French; Gr, grade; R+S+, Root present, suffix present; R+S−, Root present, suffix absent; R−S+, Root absent, suffix present; R−S−, Root absent, suffix absent.

Figure 1 presents the mean percentages of correct responses for word stimuli. French children performed more accurately than English children (95.61 and 75.97%, respectively), *F*_1(1, 71)_ = 49.41, *p* < 0.001, η^2^_*p*_ = 0.41, *F*_2(1, 224)_ = 119.74, *p* < 0.001, η^2^_*p*_ = 0.35. There was a main effect of suffix, *F*_1(1, 71)_ = 23.00, *p* < 0.001, η^2^_*p*_ = 0.25, *F*_2(1, 224)_ = 3.77, *p* = 0.05, η^2^_*p*_ = 0.02, and a root by suffix interaction in the analysis by participants only, *F*_1(1, 71)_ = 3.35, *p* = 0.04, η^2^_*p*_ = 0.06, *F*_2(1, 224)_ = 2.50, *p* = 0.11. As the root by suffix by language interaction was also significant (marginally so, by items), *F*_1(1, 71)_ = 18.51, *p* < 0.001, η^2^_*p*_ = 0.21, *F*_2(1, 224)_ = 3.20, *p* = 0.07, η^2^_*p*_ = 0.02, we examined this interaction in each group separately.

In the UK group, the root by suffix interaction was significant (marginally so, by items), *F*_1(1, 39)_ = 18.19, *p* < 0.001, η^2^_*p*_ = 0.34, *F*_2(1, 112)_ = 2.88, *p* = 0.08 η^2^_*p*_ = 0.03, indicating that while the presence of a suffix had no impact on accuracy when a root was present (R+S+: 76.03%, R+S−: 77.23%), it improved accuracy when there was no root (R−S+: 79.81%, R−S−: 70.81%). The effects were not significant in French.

#### Latencies

**Figure 2:**
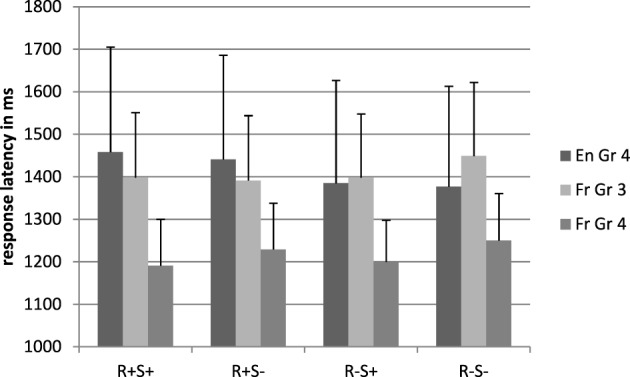
**Response latencies in the lexical decision task, real word conditions**. En, English; Fr, French; Gr, grade; R+S+, Root present, suffix present; R+S−, Root present, suffix absent; R−S+, Root absent, suffix present; R−S−, Root absent, suffix absent.

The mean latences for word stimuli.are reported in Figure 2. The French children responded faster than the English children [respectively, 1217 and 1415 ms, *F*_1(1, 71)_ = 4.71, *p* = 0.03, η^2^_*p*_ = 0.06, *F*_2(1, 224)_ = 42.90, *p* < 0.001, η^2^_*p*_ = 0.16]. There was a main effect of root [respectively, 1350 vs. 1317 ms, *F*_1(1, 71)_ = 3.94, *p* = 0.05, η^2^_*p*_ = 0.05 *F*_2(1, 224)_ = 4.32, *p* = 0.04, η^2^_*p*_ = 0.02. In addition, the root by language interaction was significant *F*_1(1, 71)_ = 9.56, *p* = 0.003, η^2^_*p*_ = 0.05, *F*_2(1, 224)_ = 4.32, *p* = 0.04, η^2^_*p*_ = 0.02]. Comparison showed a significant effect in English only, with the presence of a root slowing down latencies [R+: 1449 ms, R−: 1381 ms, *F*_1(1, 71)_ = 12.32, *p* = 0.001, η^2^_*p*_ = 0.23, *F*_2(1, 112)_ = 6.28, *p* = 0.01, η^2^_*p*_ = 0.05].

The suffix by language interaction was marginally significant by participants and non-significant by items, *F*_1(1, 71)_ = 3.33, *p* = 0.07, η^2^_*p*_ = 0.05, *F*_2(1, 224)_ = 2.19, *p* = 0.14, η^2^_*p*_ = 0.01. The presence of a suffix speeded up word recognition in French [1195 vs. 1239 ms, *F*_1(1, 29)_ = 4.42, *p* = 0.04, η^2^_*p*_ = 0.13, *F*_2(1, 112)_ = 3.22, *p* = 0.07, η^2^_*p*_ = 0.03] but not in English (1421 vs. 1409 ms), although it should be noted that this finding did not generalize across items.

### Pseudoword condition

#### Accuracy

**Figure 3:**
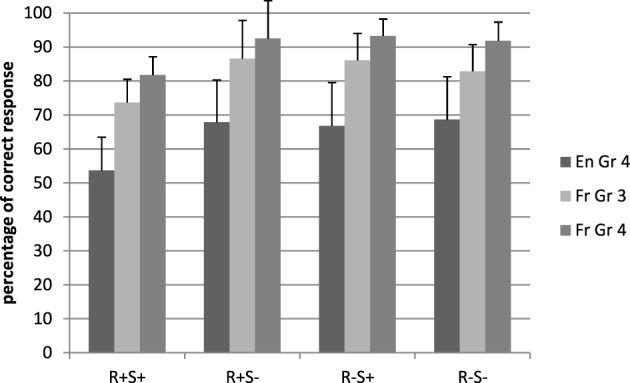
**Mean percentage of correct responses in the lexical decision task, pseudoword conditions**. En, English; Fr, French; Gr, grade; R+S+, Root present, suffix present; R+S−, Root present, suffix absent; R−S+, Root absent, suffix present; R−S−, Root absent, suffix absent.

The mean percentages of correct responses are displayed in Figure 3. French children responded more accurately than UK children [respectively, 98.8 and 64% correct, *F*_1(1, 71)_ = 31.22, *p* < 0.001, η^2^_*p*_ = 0.31, *F*_2(1, 224)_ = 38.80, *p* < 0.001, η^2^_*p*_ = 0.28]. There was a main effect of root, *F*_1(1, 71)_ = 40.58, *p* < 0.001, η^2^_*p*_ = 0.36, *F*_2(1, 224)_ = 24.24, *p* < 0.001, η^2^_*p*_ = 0.10, and an interaction between suffix and language, *F*_1(1, 71)_ = 35.35, *p* < 0.001, η^2^_*p*_ = 0.33, *F*_2(1, 224)_ = 3.80, *p* = 0.05, η^2^_*p*_ = 0.02. The root by suffix by language interaction was also significant by participants but not by items, *F*_1(1, 71)_ = 39.12, *p* < 0.001, η^2^_*p*_ = 0.36, *F*_2_ < 1. For completeness, simple effects were used to investigate the interaction by participants further but it should be noted that the interaction did not generalize across items.

For the UK children, there were significant main effects of root, *F*_1(1, 42)_ = 27.16, *p* < 0.001, η^2^_*p*_ = 0.39, *F*_2(1, 112)_ = 11.52, *p* < 0.001, η^2^_*p*_ = 0.09, and suffix, *F*_1(1, 42)_ = 25.80, *p* < 0.001, η^2^_*p*_ = 0.38, *F*_2(1, 112)_ = 19.60, *p* < 0.001, η^2^_*p*_ = 0.15. There was also a root by suffix interaction, *F*_1(1, 42)_ = 22.95, *p* < 0.001, η^2^_*p*_ = 0.35, *F*_2(1, 112)_ = 7.40, *p* = 0.008, η^2^_*p*_ = 0.06, revealing that the effect of root was significant only when there was also a suffix present, with this combination of root plus suffix reducing pseudoword accuracy.

For French children, there were main effects of root, *F*_1(1, 39)_ = 16.95, *p* < 0.001, η^2^_*p*_ = 0.37, *F*_2(1, 112)_ = 13.27, *p* < 0.001, η^2^_*p*_ = 0.11 and suffix, *F*_1(1, 39)_ = 15.82, *p* < 0.001, η^2^_*p*_ = 0.35, *F*_2(1, 112)_ = 9.46, *p* = 0.003, η^2^_*p*_ = 0.08. The root by suffix interaction was significant, *F*_1(1, 39)_ = 17.71, *p* < 0.001, η^2^_*p*_ = 0.38, *F*_2(1, 112)_ = 16.93, *p* < 0.001, η^2^_*p*_ = 0.13, and, as for the UK group, the negative effect of the root only occurred when there was also a suffix present.

In all, the morphemic effects are similar in both languages but this interaction indicates that the effects (by participants) appear stronger in the UK children.

#### Latencies

**Figure 4:**
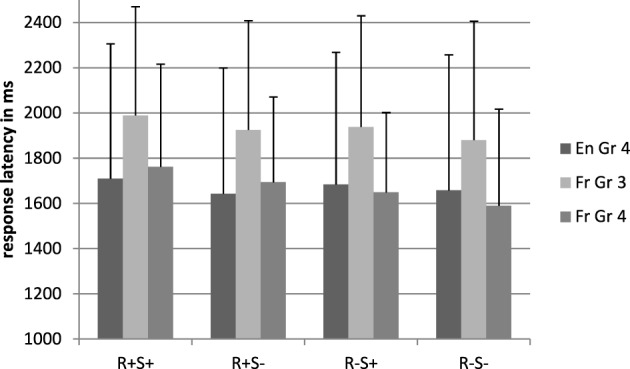
**Response latencies in the lexical decision task, pseudoword conditions**. En, English; Fr, French; Gr, grade; R+S+, Root present, suffix present; R+S−, Root present, suffix absent; R−S+, Root absent, suffix present; R−S−, Root absent, suffix absent.

Figure 4 shows the mean latencies for the pseudoword conditions. There was a main effect of root, *F*_1(1, 71)_ = 6.37, *p* = 0.014, η^2^_*p*_ = 0.08, *F*_2(1, 224)_ = 13.96, *p* < 0.001, η^2^_*p*_ = 0.06, and this effect interacted significantly with language, *F*_1(1, 71)_ = 5.18, *p* = 0.03, η^2^_*p*_ = 0.07, *F*_2(1, 224)_ = 6.08, *p* = 0.01, η^2^_*p*_ = 0.04. The negative impact of the root was present in French only [1727 vs. 1619 ms, *F*_1(1, 29)_ = 9.34, *p* = 0.005, η^2^_*p*_ = 0.24, *F*_2(1, 112)_ = 16.60, *p* < 0.001, η^2^_*p*_ = 0.13].

The suffix by language interaction was also significant by participants only, *F*_1(1, 71)_ = 7.03, *p* = 0.01, η^2^_*p*_ = 0.09, *F*_2(1, 224)_ = 1.21, *p* = 0.27, η^2^_*p*_ = 0.02. Across participants, this indicated that the presence of suffixes slowed down responses in the French group only [respectively, 1705 and 1681 ms, *F*_1(1, 29)_ = 4.64, *p* = 0.04, η^2^_*p*_ = 0.14, *F*_2(1, 112)_ = 4.17, *p* = 0.04, η^2^_*p*_ = 0.04].

### Summary of main results, part 1

In sum, sensitivity to morpheme units differed across languages in processing words, while patterns of response were more comparable for pseudoword processing.

Concerning words, the presence of a root slowed word recognition in English only. The presence of a suffix was only beneficial for English accuracy in the absence of a root. In French, the pattern was different: the presence of a suffix led to faster word recognition.

In pseudoword processing, across languages, reduced accuracy was observed when a pseudoword contained both a root and a suffix, although this effect was somewhat stronger in English. Only the French children showed latency effects, with the presence of either a root or a suffix leading to slower responses.

As the French children were younger than the UK children, a second analysis was conducted to match chronological age rather than school level.

## Results part II: chronological age matched comparison

The results of the chronological age matched children are displayed in Figures 1–4.

### Word condition

#### Accuracy

The French children performed more accurately than the UK children [respectively, 89.99 vs. 75.97%, *F*_1(1, 71)_ = 23.12, *p* < 0.001, η^2^_*p*_ = 0.78, *F*_2(1, 224)_ = 261.90, *p* < 0.001, η^2^_*p*_ = 0.70], in spite of having received a year less of schooling. There was a main effect of suffix, *F*_1(1, 71)_ = 34.60, *p* < 0.001, η^2^_*p*_ = 0.33, *F*_2(1, 224)_ = 6.07, *p* = 0.01, η^2^_*p*_ = 0.03, a suffix by root interaction (marginal by items), *F*_1(1, 71)_ = 8.01, *p* = 0.006, η^2^_*p*_ = 0.10, *F*_2(1, 224)_ = 2.85, *p* = 0.09, and the root by suffix by language interaction was significant by participants only, *F*_1(1, 71)_ = 11.37, *p* < 0.001, η^2^_*p*_ = 0.14, *F*_2(1, 224)_ = 1.48, *p* = 0.23. For completeness, the interaction by participants was followed up using simple effects for each language group separately, although it should be noted that the interaction does not generalize across items.

The UK group showed a main effect of suffix in the analysis by participants, *F*_1(1, 41)_ = 21.70, *p* < 0.001, η^2^_*p*_ = 0.34, *F*_2(1, 112)_ = 2.09, *p* = 0.15 and an interaction between suffix and root (marginal by items), *F*_1(1, 41)_ = 18.19, *p* < 0.001, η^2^_*p*_ = 0.30, *F*_2(1, 112)_ = 3.54, *p* = 0.07, η^2^_*p*_ = 0.07, indicating that suffixes improved word recognition accuracy only when there was no root.

For the French group, only the main effect of suffix was significant, *F*_1(1, 29)_ = 13.54, *p* < 0.001, η^2^_*p*_ = 0.32, *F*_2(1, 112)_ = 4.63, *p* = 0.03, η^2^_*p*_ = 0.04: words with a suffix were recognized more accurately than words without suffix (92.20 vs. 87.50%, respectively).

#### Latencies

There was no main effect of language but the root by language interaction was significant, *F*_1(1, 71)_ = 11.43, *p* = 0.001, η^2^_*p*_ = 0.14, *F*_2(1, 224)_ = 2.82, *p* = 0.03, η^2^_*p*_ = 0.14: roots increased response latencies in the UK group only, *F*_1(1, 41)_ = 12.32, *p* = 0.001, η^2^_*p*_ = 0.23, *F*_2(1, 112)_ = 6.28, *p* = 0.01, η^2^_*p*_ = 0.05.

### Pseudoword condition

#### Accuracy

French children were more accurate than UK children [82.30 vs. 64% correct, respectively, *F*_1(1, 71)_ = 14.17, *p* = 0.01, η^2^_*p*_ = 0.17, *F*_2(1, 224)_ = 8.96, *p* = 0.003, η^2^_*p*_ = 0.04]. While there was no main suffix effect, the suffix by language interaction was significant by participants only, *F*_1(1, 71)_ = 33.16, *p* < 0.001, η^2^_*p*_ = 0.32. The root by suffix by language interaction was also significant only in the analysis by participants, *F*_1(1, 71)_ = 52.12, *p* < 0.001, η^2^_*p*_ = 0.42. Although this effect does not generalize across items, simple effects were used to understand the interaction by participants.

In the UK group, there were main effects of root, *F*_1(1, 42)_ = 27.16, *p* < 0.001, η^2^_*p*_ = 0.39, *F*_2(1, 112)_ = 11.52, *p* < 0.001, η^2^_*p*_ = 0.09, and suffix, *F*_1(1, 42)_ = 25.80, *p* < 0.001, η^2^_*p*_ = 0.38, *F*_2(1, 112)_ = 19.60, *p* < 0.001, η^2^_*p*_ = 0.15, and an interaction between root and suffix, *F*_1(1, 42)_ = 22.95, *p* < 0.001, η^2^_*p*_ = 0.35, *F*_2(1, 112)_ = 7.80, *p* = 0.008, η^2^_*p*_ = 0.06, indicating that the combination of a root and a suffix decreased accuracy relative to other pseudowords.

In the French group, main effects of root, *F*_1(1, 29)_ = 12.17, *p* = 0.002, η^2^_*p*_ = 0.30, *F*_2(1, 112)_ = 3.68, *p* = 0.05, η^2^_*p*_ = 0.04, and suffix, *F*_1(1, 29)_ = 11.64, *p* < 0.001, η^2^_*p*_ = 0.29, *F*_2(1, 112)_ = 4.85, *p* = 0.03, η^2^_*p*_ = 0.04, were also observed, as well as an interaction between root and suffix, *F*_1(1, 29)_ = 30.17, *p* < 0.001, η^2^_*p*_ = 0.51, *F*_2(1, 112)_ = 11.42, *p* < 0.001, η^2^_*p*_ = 0.09. The interaction revealed reduced accuracy for the combination of a root and a suffix compared to other pseudowords.

This inspection of the data reveals that the suffix by root by group interaction (by participants) reflects the fact that the effects were stronger in French than in English.

#### Latencies

UK children responded faster than French children [respectively, 1674 and 1934 ms, *F*_1(1, 71)_ = 6.77, *p* = 0.04, η^2^_*p*_ = 0.06, *F*_2(1, 224)_ = 146.08, *p* < 0.001, η^2^_*p*_ = 0.40]. Across groups, response latencies were longer when a suffix was present [respectively, 1831 and 1777 ms, *F*_1(1, 71)_ = 6.87, *p* = 0.011, η^2^_*p*_ = 0.09, *F*_2(1, 224)_ = 7.31, *p* = 0.007, η^2^_*p*_ = 0.03].

### Summary of main results, part II

As in the comparison by grade level, there was indication of differential group sensitivity to morpheme units in word recognition but a similar pattern of pseudoword processing across languages.

For word recognition, only the UK children showed increased latencies when a root was present. Accuracy among the French children showed a higher degree of sensitivity to suffixes (regardless of whether a root was present or not).

For pseudoword processing, the effects were the same across languages: the presence of a suffix in a pseudoword slowed responses and the combination of a root plus a suffix reduced accuracy.

## Discussion

Current models of reading development highlight cross-linguistic variation in naming accuracy in relation to early orthographic decoding (e.g., Ziegler and Goswami, 2005). However, these models do not offer an account of whether or not cross-linguistic effects operate on morphological processing during visual word recognition. The present study examined the extent to which morphemic effects in lexical access are universal or whether such effects can be modulated by language specificities during development.

For this purpose two comparable sets of lexical decision stimuli that manipulated the presence of component morphemes were presented to groups of French- and English-speaking children. As schooling starts 1 year earlier in the UK as compared to France, performance was first compared using a schooling match (Grades 4 in France and the UK), and in a second comparison, a chronological age match (Grade 3 in France and Grade 4 in the UK; both aged 8 years).

The data clearly indicate the importance of roots and suffixes for both language groups. Although the precise pattern differed, both groups were sensitive to the presence a suffix within words—either a genuine suffix or a suffix-like ending—which is consistent with the importance of suffixes as orthographic patterns. For pseudowords, the combination of a root with a suffix interfered with accurate processing in both languages. A tendency to slower responses was also observed when a pseudoword contained only a suffix, although this effect was clearer in French and present from Grade 3 onwards.

Cross-linguistic differences were also apparent, although some interaction effects were significant in the by-participants analysis only. In English, the presence of roots slowed down visual word recognition. Specific attention was given to the R+S+ vs. R+S- comparison, as these correspond respectively to the morphological and orthographic control conditions that are typically used in the literature on morphological decomposition in visual word recognition (see for example, Feldman et al., 2002; Casalis et al., 2009, for developmental studies). Interestingly, faster word recognition was observed when a suffix was present in the French analysis but not in the English analysis. This suggests a more specifically *morphological* sensitivity in French, whereas the results indicated sensitivity to embedded words in English, since roots were mostly free-standing words.

In English, suffixes only affected the accuracy of word recognition in the absence of a root; whereas, in French, suffixes generally led to faster word recognition and, for the older Grade 4 group, improved accuracy only when combined with a root (i.e., the R+S+ real derivations, e.g., farmer). This latter effect of school grade in French suggests that reading skills and/or language proficiency has an impact on suffix processing.

A detrimental effect of the root was observed in English only. This effect was not apparent in French as the impact of the root produced only facilitation effects among French children. This cross-language discrepancy may derive from the fact that, in most cases, roots corresponded to whole words in English (41 out 58 items), whereas this was less true of French (25 out 58 items). This would be consistent with Nation and Cocksey's (2009) finding of an automatic semantic activation of embedded words among English-speaking 7-year-olds. Therefore, the inhibition effect observed in the present study may reflect processing costs associated with identification of the root and competition with whole word processing. Indeed, a striking finding is that the inhibition effect in English is observed in both R+S− words, which may be considered to be orthographic control items, and R+S+, which are derived forms. Morphological priming studies report only facilitation effects, both among skilled readers of English (e.g., Rastle and Davis, 2008) and developing readers of French (Quémart et al., 2011). Minimally, then the inhibition effects observed here indicate that young English-speaking readers are sensitive to embedded word units in visual word recognition. While higher frequency embedded words in English might have favored an inhibition effect in English, the languages did not differ significantly in this respect in either the R+S+ or R+S = conditions although it should be noted that the outcome was marginal (*p* = 0.07) in the R+S− condition. While French and English stimuli were statistically matched in terms of frequency, French words tended to be slightly longer than English words. Although the difference was less than 1 letter on average, this could potentially have led French children to show more reliance on a decomposition strategy for word processing. Another source of difference lies in the fact that some suffixes have been repeated, leading potentially to an increased sensitivity to morphological decomposition. Note that slightly more repetition occurred in English (-er) than French.

Strong effects of morpheme units were found in pseudoword processing where the combination of both root and suffix led to an increasing rate of errors. This result is in line with previous research, suggesting that young readers rely on morpheme units when they have to process an unknown word (Burani et al., 2002; Quémart et al., 2012). At the same time, linguistic variation also came into play as the beneficial effects of suffixes, in particular, were stronger in French pseudoword processing.

A methodological difficulty when comparing children from different countries is that such a comparison goes beyond differences in native language. A first issue is that schooling starts during the fifth year in UK while it starts during the sixth year in France. This was dealt with by performing two separates analyses: one based on a school-level matched design, with French children being older than UK children; and the other based on a chronological-age matched design, with the UK children having experienced a year more of schooling. It was not possible to achieve a perfect matching between the groups as the English-speaking children were less accurate than the French children regardless of the method of matching groups. In contrast, the UK children exhibited slower latencies in word processing than the older Grade 4 French children in the first analysis but were faster at pseudoword processing than the Grade 3 French children in the second analysis. A second issue is connected to the school curriculum, particularly in relation to the teaching of reading and morphology. Across languages, our participants all came from schools adopting a mixed method approach to reading instruction: whole-word and phonics. In France, morphological structure is explicitly taught at Grade 4 and, in the Scottish education system that the UK children experienced, intensive instruction about derivational affixes begins in Grades 3 or 4 as part of spelling instruction. Further studies should address instructional issues in a more systematic way. Our study was a first attempt to directly compare the use of morphological units across languages. It will therefore be necessary to extend this work to larger samples as well as other languages.

In terms of group differences in word processing, French children were always more accurate, and were faster only when they were older (schooling matching); in pseudoword processing, French children again always responded more accurately, but responded more slowly when they were matched on age (with less schooling). Note that the difference may be explained by the fact that the French pseudowords were almost one letter longer than the English pseudowords. However, it is possible that the additional year of schooling experienced by the UK children may also have contributed to their faster pseudoword reaction times. Beyond these group differences, both analyses yielded quite similar patterns of results. However, the slight differences that emerged between Part 1 and Part 2 reveal that morphological processing develops during schooling. More specifically, the presence of a root slowed down latencies for fourth graders only (UK), and there were more indication of a suffix benefit among French fourth graders than French third graders.

Thus, our study demonstrates that developing readers make use of morphology when recognizing familiar words and when processing new words. In a previous study, Duncan et al. (2009) compared English and French morphological awareness in relation to derivation with suffixes. The results clearly showed that the UK children were outperformed by the French children when they had to manipulate morpheme units explicitly. Note that sensitivity to morphemes, as assessed by a relational judgment task, was found to be similar in both groups. These results were interpreted with reference to the importance of morphological structure in French. It is therefore interesting to note that, in the present study, UK children make use of morphemes during lexical access, even though overall they were less accurate at this than French children and were less sensitive to true derivations (R+S+ words). This outcome aligns with two conclusions: first, morphemes may be used in word and pseudoword processing regardless of GPC transparency; and second, when confronted with a rich morphological system, children may develop morphological knowledge faster and acquire a more finely-tuned sensitivity to written morphology.

In conclusion, research on reading acquisition reflects a growing interest in morphological processing, as once children have completed the first phases of reading acquisition they are confronted by a growing number of long and derived words. Previous research on phonological coding in reading aloud has pointed to the importance of cross-language variation in the nature and speed of acquisition of GPC, and was formalized in the PGST. Our intention was to begin the process of examining morphological processing in visual word recognition within a similar framework. The languages under investigation differed in terms of orthographic depth, with English being more opaque than French, and morphological productivity, with French being morphologically richer than English. The first aim was to examine whether morphology was generally used by developing readers in Grades 3 and 4. A main result was that children make use of morphemic information in both languages confirming our first hypothesis of the relevance of morphology in reading development in both opaque and more transparent alphabetic orthographies. The second aim was to assess the importance of two factors expected to be influential, namely, orthographic depth and morphological prevalence/transparency. Both aspects could be contrasted in English and French in opposing directions, with the English orthography being more opaque and French morphology being richer. One key question was therefore whether the utility of morphemes in resolving the greater inconsistency in English increased sensitivity in this group beyond the level expected by the influence of morphological productivity/transparency. Results indicated stronger morphological effects in French, confirming our hypothesis that morphological richness will outweigh orthographic depth at least in alphabetic writing systems.

### Conflict of interest statement

The authors declare that the research was conducted in the absence of any commercial or financial relationships that could be construed as a potential conflict of interest.
