# Supplementary material for: How language affects children's use of derivational morphology in visual word and pseudoword processing: evidence from a cross-language study
Source: Front Psychol. 2015 Apr 16;6:452. doi: 10.3389/fpsyg.2015.00452 (PMC4399200; doi:10.3389/fpsyg.2015.00452)
Supplement: Supplementary file 1 [file DataSheet1.DOCX]

**Appendix**

Detailed list of the English and French targets

| **English Words** | | | | | |
| --- | --- | --- | --- | --- | --- |
| **R+S+** | | **R+S-** | | **R-S+** | **R-S-** |
| **word** | **root** | **word** | **root** | **word** | **word** |
| pressure | press | spinach | spin | celery | stomach |
| cookery | cook | vanilla | van | cabbage | gorilla |
| baggage** | bag | menace | men | royal | terrace |
| tidal* | tide | turnip | turn | duty | tulip |
| nosy* | nose | tuna* | tune | jealous | sofa |
| famous* | fame | madam | mad | sausage | album |
| passage | pass | palace | pal | serious | bureau |
| nervous* | nerve | denim | den | rascal | victim |
| postal | post | carrot** | car | leisure | parrot |
| failure | fail | costume | cost | pony | perfume |
| bony* | bone | tuba* | tube | obvious | cola |
| joyous | joy | tennis** | ten | cymbal | crisis |
| signal | sign | lentil | lent | crystal | fossil |
| central* | centre | wallop | wall | nasty | circus |
| dirty | dirt | pasta | past | garage | zebra |
| bandage | band | lettuce** | Let | measure | husband |
| moisture | moist | pencil* | pence | voyage | hermit |
| wreckage | wreck | surface | surf | future | furnace |
| pleasure* | please | legend | leg | precious | biscuit |
| gracious* | grace | minus* | mine | pepper | walrus |
| robber** | rob | dollop | doll | father | burrow |
| digger** | dig | robot* | robe | sister | narrow |
| farmer | farm | barrow** | bar | glitter | bishop |
| roller | roll | yellow | yell | murder | rabbit |
| duster | dust | window | wind | spider | meadow |
| driver* | drive | fellow | fell | tiger | cactus |
| piper* | pipe | pilot* | pile | weather | visit |
| teacher | teach | shadow* | shade | hoover | nephew |
| hopper** | hop | pillow | pill | treasure | follow |
| **French Words** | | | | | |
| **R+S+** | | **R+S-** | | **R-S+** | **R-S-** |
| **word** | **root** | **word** | **root** | **word** | **word** |
| berceau* | berce | crique | cri | auteur | pédale |
| chasseur* | chasse | bourrique* | bourrer | bocal | toque |
| coléreux* | colère | tactique | tact | bureau | persil |
| coûteux | coût | salive* | Sale | cadeau | ivoire |
| douceur* | doux | orgueil | orgue | cahier | rubrique |
| douteux* | doute | nuque | nu | carnaval | cigale |
| equipage* | équipe | univers | uni | cerveau | lessive |
| fermier* | ferme | tirelire* | tirer | chameau | claque |
| garage* | garer | plaque* | plat | corbeau | angoisse |
| grillage* | grille | capitale | cap | cristal | brousse |
| hauteur | haut | caresse | car | dernier | flaque |
| joueur | joue | pinson | pin | entier | poison |
| journal | jour | coque | coq | étage | panique |
| lainage* | laine | casque | cas | généreux | accueil |
| national | nation | masque | mas | gibier | banque |
| olivier* | olive | prison* | prise | hôpital | phoque |
| paysage | pays | boutique | bout | février | risque |
| Pêcheur* | pêche | disque | dis | jumeau | pique |
| plateau | plat | envers* | envie | marteau | attaque |
| plumage* | plume | barque | bar | meilleur | remarque |
| policier* | police | mousse | mou | ménage | mémoire |
| pommier* | pomme | fauteuil | faute | métal | saison |
| rivage* | rive | marque* | mare | métier | époque |
| signal* | signe | écureuil* | écurie | plage | conseil |
| tonneau* | tonne | raison* | raie | rumeur | caisse |
| troupeau* | troupe | cirque* | cire | sérieux | manque |
| verbal* | verbe | musique* | muse | vaisseau | navire |
| village* | ville | poisson | pois | vénéneux | travers |
| voyageur* | voyage | abricot | abri | vertical | classe |

R+S-: Root present, suffix absent

R+S+: Root present, suffix present

R-S-: Root absent, suffix absent

R-S+: Root absent, suffix present

*: the base word is not a free standing root.

**: the base word is modified in the derived form by doubling the consonant.

Detailed list of the English and French pseudowords

| **English Pseudowords** | | | | | | |
| --- | --- | --- | --- | --- | --- | --- |
| **R+S+** | | **R+S-** | | **R-S+** | **R-S-** | |
| **pseudoword** | **root** | **pseudoword** | **root** | **pseudoword** | **pseudoword** | |
| trickure | trick | trimach | trim | drealure | trenach | |
| waitery | wait | potilla | pot | muvery | tadilla | |
| battage | bat | gumace | gum | hettage | collace | |
| wifal* | wife | curlip | curl | cayal | cunip | |
| namy* | name | hosa* | hose | raby | mava | |
| holous* | hole | fatam | fat | foadous | astum | |
| Tellage | tell | jarace | jar | reanage | cuneau | |
| falsous* | false | matim | mat | suvious | gerlim | |
| pondal | pond | sunnot** | sun | vosnal | seggot | |
| joinure | join | girlume | girl | ceinure | berlume | |
| pagy* | page | fuma* | fume | josy | vema | |
| bayous | Bay | wettis** | wet | erdious | glavis | |
| bombal | bomb | toldil | told | syndal | porril | |
| fibral* | fibre | kissop | kiss | gryspal | bilbus | |
| landy | land | firma | firm | cusky | volna | |
| cornage | corn | fedduce** | fed | temage | birtand | |
| weirdure | weird | gorgil* | gorge | paiture | perdit | |
| chainage | chain | burnace | burn | lewage | bormace | |
| freezure* | freeze | macend | mac | lenure | fercuit | |
| spicious* | spice | firus* | fire | flocious | zernus | |
| mopper** | mop | messop | mess | sebber | nallow | |
| binner** | bin | capot* | cape | pesher | hannow | |
| gasper | gasp | tinnow** | tin | tisker | hethop | |
| misser | miss | runnow** | run | stanner | waffit | |
| gifter | gift | bulbow | bulb | gopter | ferbow | |
| braker* | brake | wonnow** | won | plager | simpus | |
| dazer* | daze | wavot* | wave | cimer | cemit | |
| coacher | coach | flamow* | flame | moacher | feshew | |
| sagger** | sag | puffow | puff | wooker | sullow | |
| **French Pseudowords** | | | | | | |
| **R+S+** | | **R+S-** | | **R-S+** | **R-S-** | |
| **pseudoword** | **root** | **pseudoword** | **root** | **pseudoword** | **pseudoword** | |
| ecolage* | école | comptale* | compte | lirtage | bedale |  |
| fraisage* | fraise | chancers | chance | mivage | gadale |  |
| normage* | norme | patinil | patin | vérage | chaders |  |
| prunage* | prune | cendril* | cendre | larpal | madil |  |
| révage* | réve | guerril* | guerre | midal | paril |  |
| rosage* | rose | tablil* | table | nondal | vamil |  |
| roulage* | rouler | comédire* | comédie | rumal | atire |  |
| sucral* | sucre | boitive* | boîte | tapinal | ovire |  |
| ciral* | cirer | baveque | bave | tenal | pagire |  |
| grossal | gros | colque* | colle | bafeau | fodive |  |
| marial | mari | comptul* | compte | ceseau | araque |  |
| ameau* | ami | cremeque | crème | mupleau | burque |  |
| glaceau* | glace | ecloque* | éclore | nupreau | esique |  |
| moucheau* | mouche | envique* | envie | sabeau | lique |  |
| routeau* | route | fièvrol | fièvre | sateau | lorque |  |
| bananeur* | banane | finique* | finir | talleau | marique |  |
| ceriseur* | cerise | fronque* | front | vitieau | nusique |  |
| denteur | dent | herbique* | herbe | nadeur | nutique |  |
| mondieur* | monde | hiverque | hiver | plimseur | prique |  |
| percheur* | perche | lainoque | laine | vebeur | rileque |  |
| banqueux* | banque | lionque | lion | cherpeux | rutrique |  |
| bruteux* | brute | plumile* | plume | fideux | soque |  |
| centreux* | centre | cyclan* | cycle | gérieux | sublique |  |
| filtreux* | filtre | fruison* | fruit | mutier | mélison |  |
| bandier* | bande | guerson* | guerre | pasier | mison |  |
| lioncier | lion | laitin | lait | risier | lifasse |  |
| rasier* | raser | gardisse* | garder | sorier | riosse |  |
| séchier | sec | nagasse* | nager | souvier | prisse |  |
| fondage | fond | campage | camp | torier | trisse |  |

*: the base word is not a free standing root.

**: the base word is modified in the derived form by doubling the consonant.
